# Laparoscopically treated pancreatic insulinoma. Case report

**Published:** 2010-05-25

**Authors:** A Miron, V Calu, C Giulea, S Fica, C Barbu, C ştefan

**Affiliations:** * Department of General Surgery, ‘Elias’ University Emergency Hospital, BucharestRomania; ** Department of Endocrinology, Diabetes and Nutrional Diseases, ‘Elias’ University Emergency Hospital, BucharestRomania

**Keywords:** pancreatic insulinoma, laparoscopic surgery

## Abstract

Usually, insulinomas are small sized, insulin secreting, benign tumors of the pancreas, and require surgical treatment. We report the case of a female patient, of 61 years old, with pancreatic insulinoma localized in the junction between the head and the istm of the pancreas, of 1,4 cm in size, which induced hypoglycemia due to endogenous insulin hypersecretion. The tumor was removed by laparoscopic enucleation in March 2009. In the postoperative period, the blood glucose level came back to normal.

## Introduction

Insulinoma is an insulin–secreting tumor, benign (over 90% of cases) or malignant, single or multiple. More than 99% of insulinomas appear in the pancreas, with an equal distribution in the head, body and tail of the gland. Most of them are adenomas, usually small–sized, under 2 cm (90%). Less than 10% of the adenomas are multiple, frequently associated with other neuroendocrine tumors, in the multiple endocrine neoplasia syndrome type Ⅰ (MEN – Ⅰ).

Insulinomas' treatment is surgical; the procedures (duodenopancreatectomy, distal pancreatectomy or enucleation) can be performed by open or laparoscopic approach.

### Case Presentation

We present the case of a female patient, of 61 years old, with Basedow disease, receiving treatment with synthetic antithyroid drugs. The patient was admitted in November 2008 in the Endocrinology unit, various tests were performed and the diagnosis of insulinoma with endogenous insulin hypersecretion was established. In January 2009, after US endoscopy and MRI were performed, a small nodule, of 1,4 cm in diameter, was discovered in the junction between the head and the istm of the pancreas, with mild bulking of the anterior pancreatic delineation. Thus, the diagnosis of a single pancreatic insulinoma was established and surgical treatment was decided and performed.

In March 2009, the patient was admitted in the Department of Surgery and laparoscopic enucleation of the tumor was performed.

### Surgical procedure

The patient was lying in dorsal decubitus position with the legs spread. We used 5 trocars: one, optic, one for retraction and three working trocars.

The stomach is grasped with atraumatic forceps and lifted. A wide opening in the gastro–colic ligament is performed by using the cautery and Ligasure

**Figure 1:**
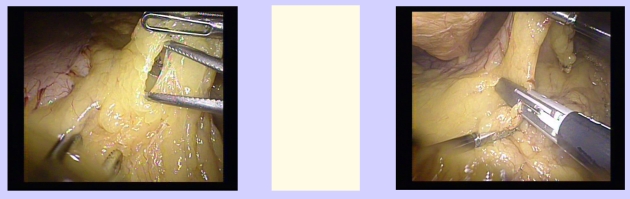
a–b: The opening of the gastro–colic ligament

The adhesions of the posterior gastric wall are severed with the scissors or hook.

**Figure 2:**
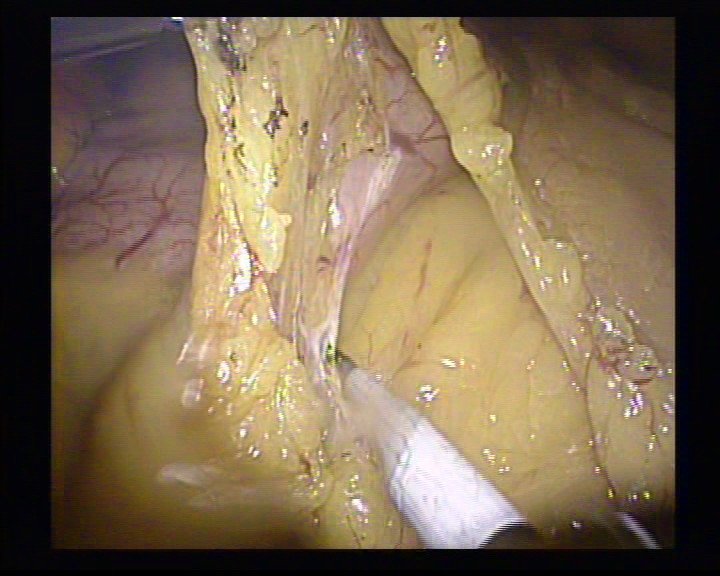
The severing of the posterior gastric wall adhesions

The stomach is lifted with a retractor. Thus, the pancreas is exposed by the elevation of the stomach and pulled downwards the colon with the atraumatic forceps. The approach is enhanced by putting the operating table in anti–Trendelenburg position and right tilt.

**Figure 3:**
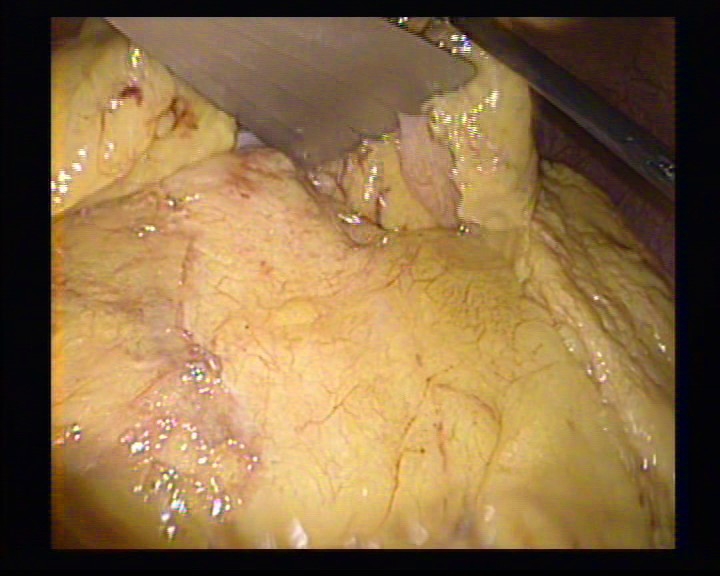
The lifting of the stomach

Once the access in the omental pouch is achieved, the dissection can be started immediately after the tumor's location is determined (directly by using US). Initial mobilization allows direct US scanning of the anterior and part of the posterior face of the pancreas.

**Figure 4:**
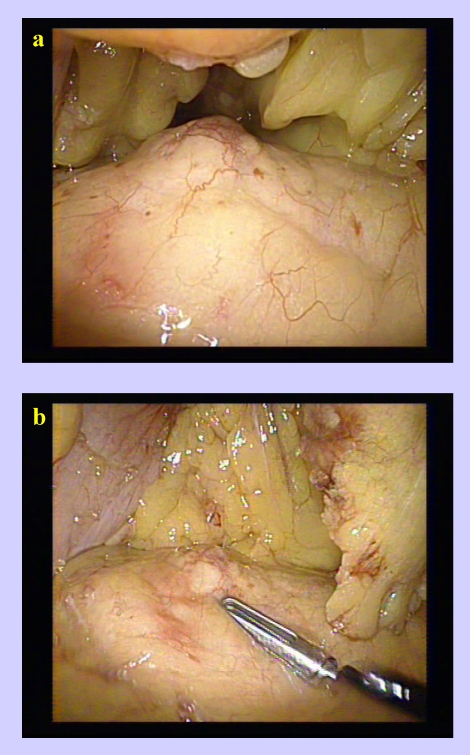
The exposure of the tumor

The enucleation is done by using the hook, bipolar forceps and Ligasure device. 

The dissection around the insulinoma is followed by a partial destruction of the normal pancreatic tissue surrounding the tumor. Thus, a groove around the tumor is created, with complete freeing on all sides from the surrounding pancreatic tissue. A special care is needed because the splenic artery is in the immediate vicinity, the dissection being performed tangent to it.

**Figure 5:**
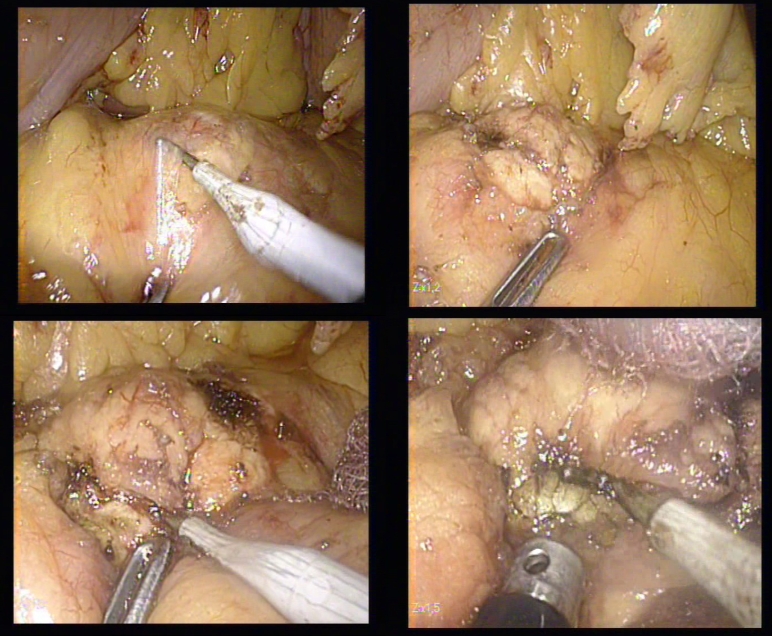
The dissection of the insulinoma using the hook

Deep and final adhesions of the tumor were difficult to access and to dissect with the hook, therefore they were sealed and divided with the Ligasure device.

**Figure 6:**
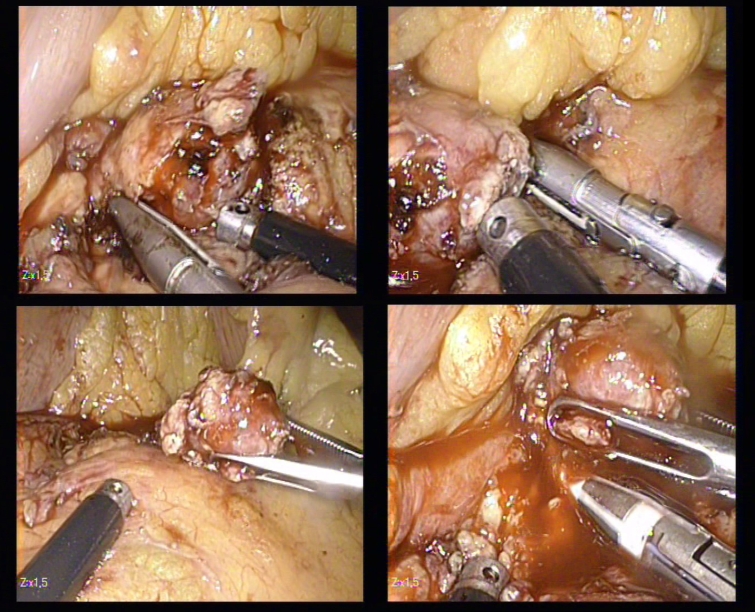
The dissection of the insulinoma using the LigaSure

After the removal of the tumor in the endo–bag, peritoneal lavage was done. The completion of hemostasis was achieved with the cautery, mild compression or sticking Surgicel.

**Figure 7:**
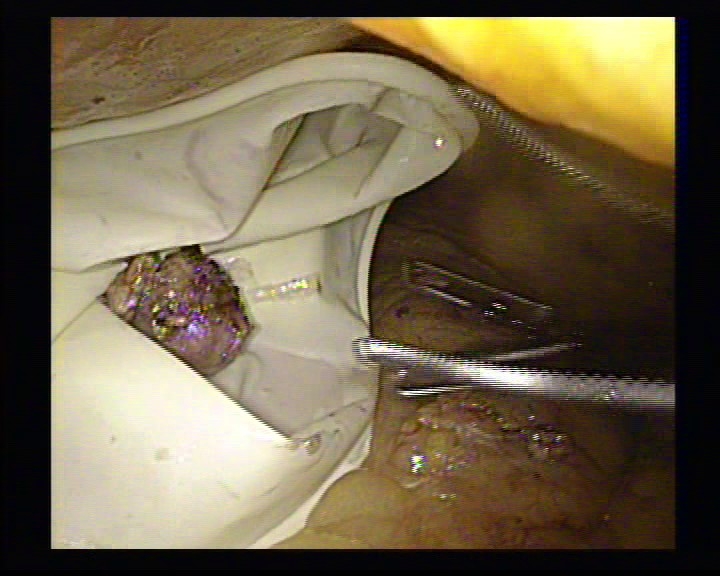
The removal of the tumor in the endo–bag

**Figure 8:**
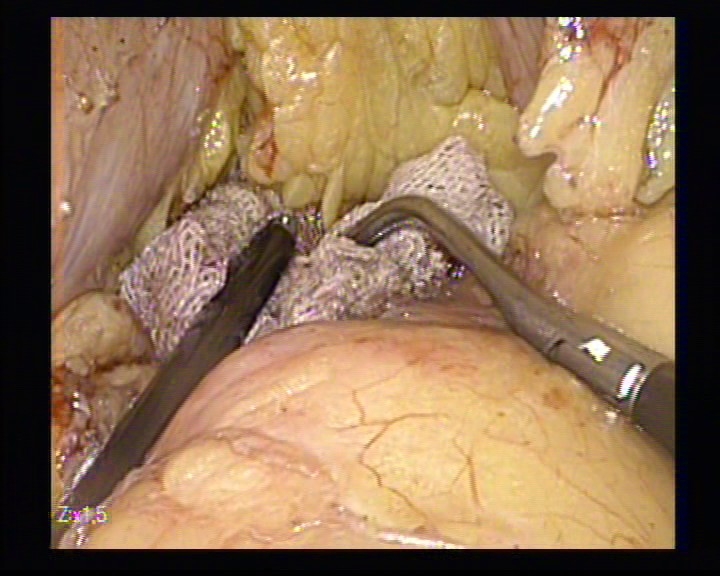
The hemostasis with mild compression

**Figure 9:**
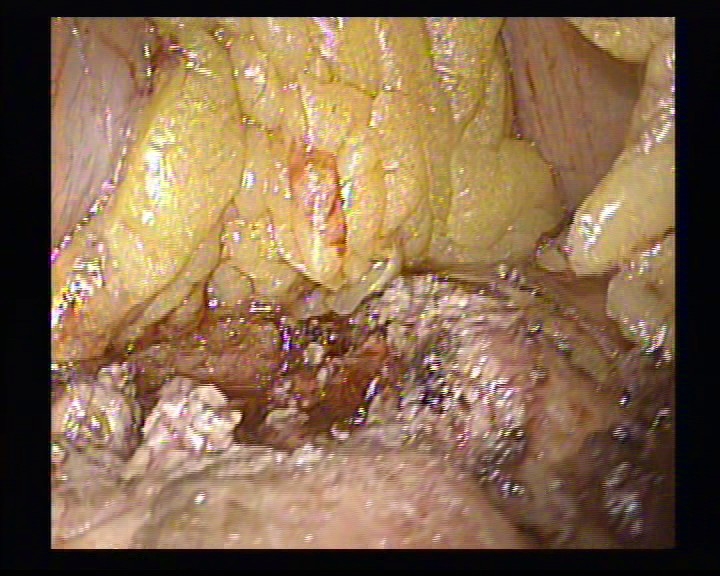
The remaining cavity

**Figure 10:**
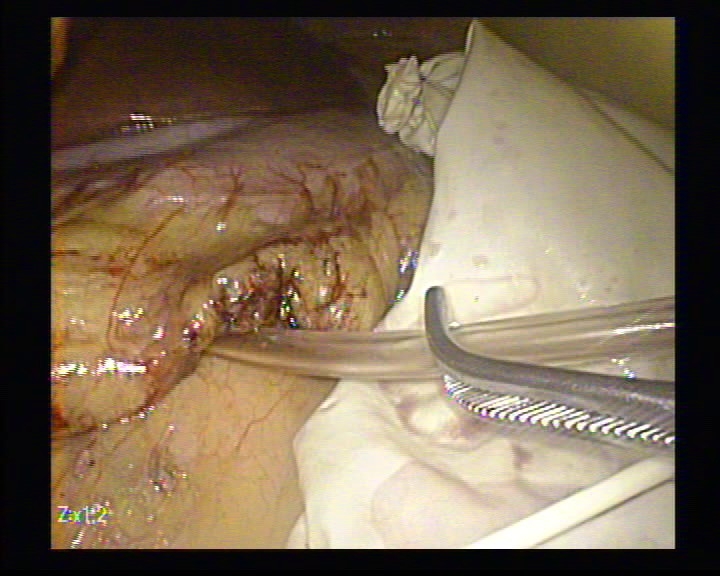
The drainage tube placed in a retrogastric position

The tumor was removed from the abdomen through a 10 mm port.

**Figure 11:**
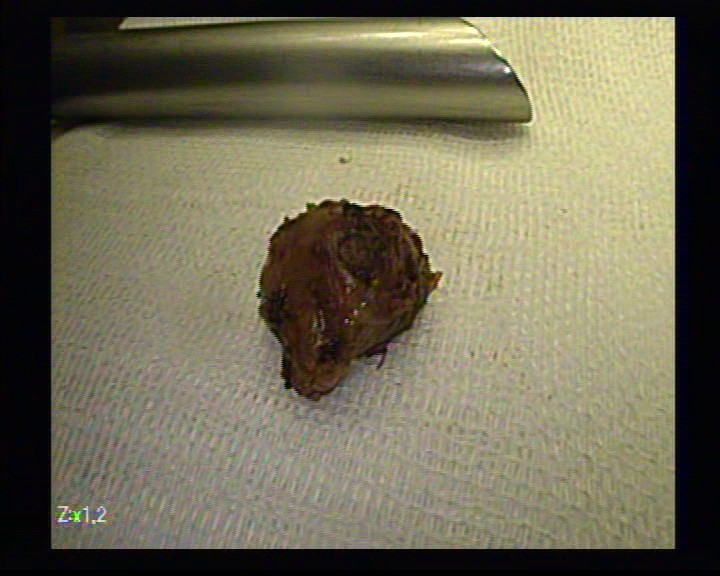
The tumor

The procedure lasted for 1h 50 min with minimal blood loss. The drainage tube was removed after 24 hours. No pancreatic leak was encountered during and after the operation, so normal and complete feeding was started.

Immediately after the procedure, a sudden increase in blood glucose level was encountered, with a maximum after 6 hours (273 mg/dl). Blood glucose level came back to normal in the first p.o.d. The patient was discharged in the third p.o.d. No postoperative complications were noticed.

The pathology report confirms the diagnosis of benign pancreatic tumor: insulinoma.

## Discussion

Pancreatic insulinoma surgical treatment usually implies either classical pancreatic resection, or, better, enucleation of the tumor [1, 2]. Laparoscopic pancreatic resection is no longer new in the field of mini–invasive surgery. [3,4]. Regarding the procedure itself, it entirely respects the principles of the open surgery.

During the operation, the action of accurately locating the tumor is essential before the enucleation. Intraoperative ultrasound, with a high rate of detection of the insulinoma, 83 – 100%, is the most sensitive method available. Laparoscopic US is already recognized as a useful tool for the intraoperative detection of the pancreatic insulinoma [5, 6, 7].

The use of Ligasure device, adequate retraction and electric dissection allowed us to remove the insulinoma without damaging the surrounding tissue.

When the tumor is close to splenic vessels or pancreatic duct, the ideal technique is represented by the ultrasound dissection devices, which have a selective effect on the tissues, preserving structures rich in collagen, like blood vessels, during the dissection of parenchyma. Avoiding a large incision for the removal of a very small tumor is a significant advantage of laparoscopic surgery. This benefit was consequently reflected in the fast, without complications, recovery of this patient.
